# Supplementary material for: Restraint of melanoma progression by cells in the local skin environment
Source: eLife. 2026 Jun 30;13:RP101974. doi: 10.7554/eLife.101974 (PMC13318302; doi:10.7554/eLife.101974)

Figure 4 - Source Data 1 - Labeled Western blot image of TWIST1

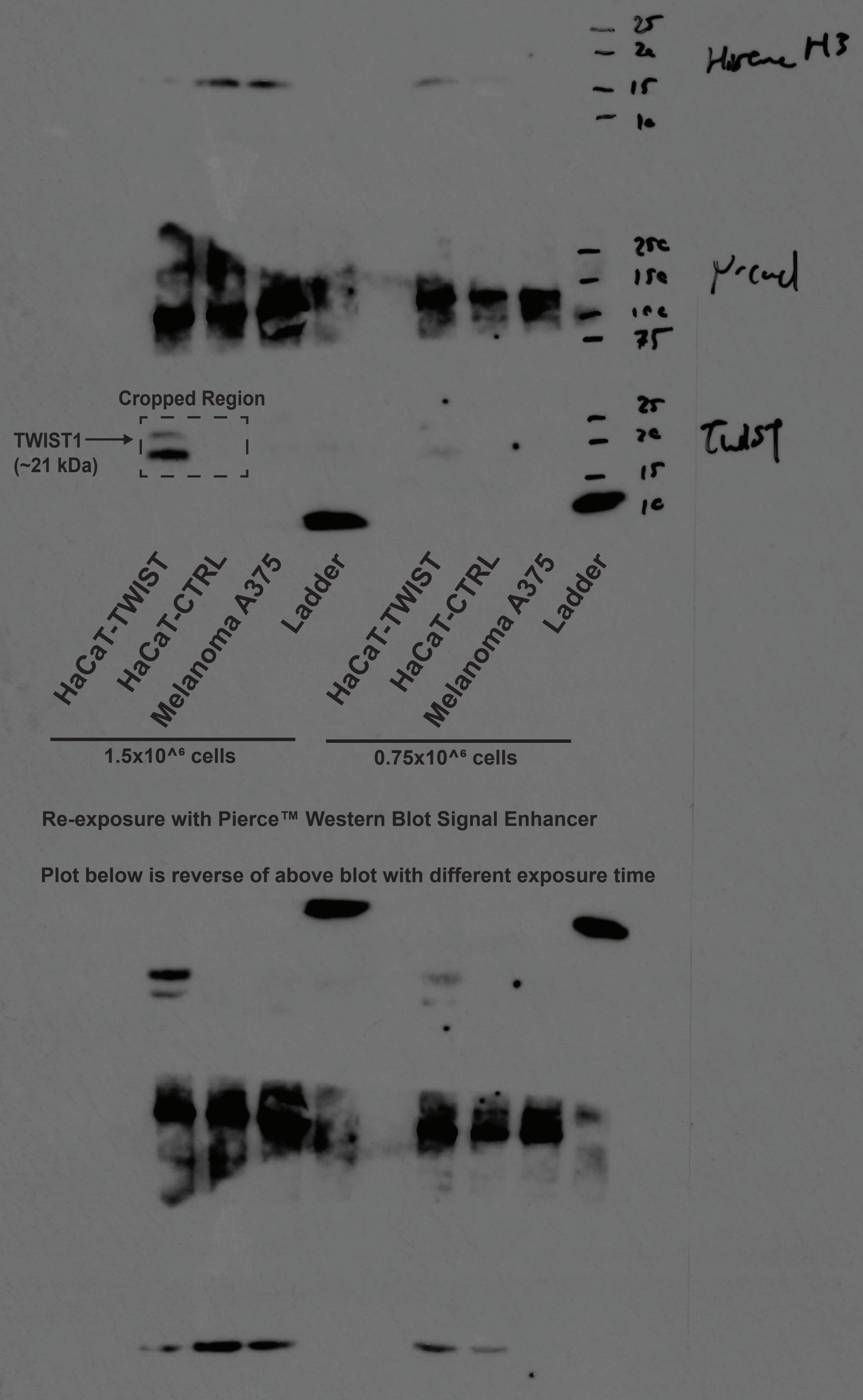

Supplement: Figure 4—source data 1. [file elife-101974-fig4-data1.zip › Figure_4C_Source_Data_1_TWIST_labled.pdf]
