# Supplementary figures and images for: Restraint of melanoma progression by cells in the local skin environment
Source: eLife. 2026 Jun 30;13:RP101974. doi: 10.7554/eLife.101974 (PMC13318302; doi:10.7554/eLife.101974)

Figure 4 - Source Data 1 - Labeled Western blot image of Actin

10/09/22

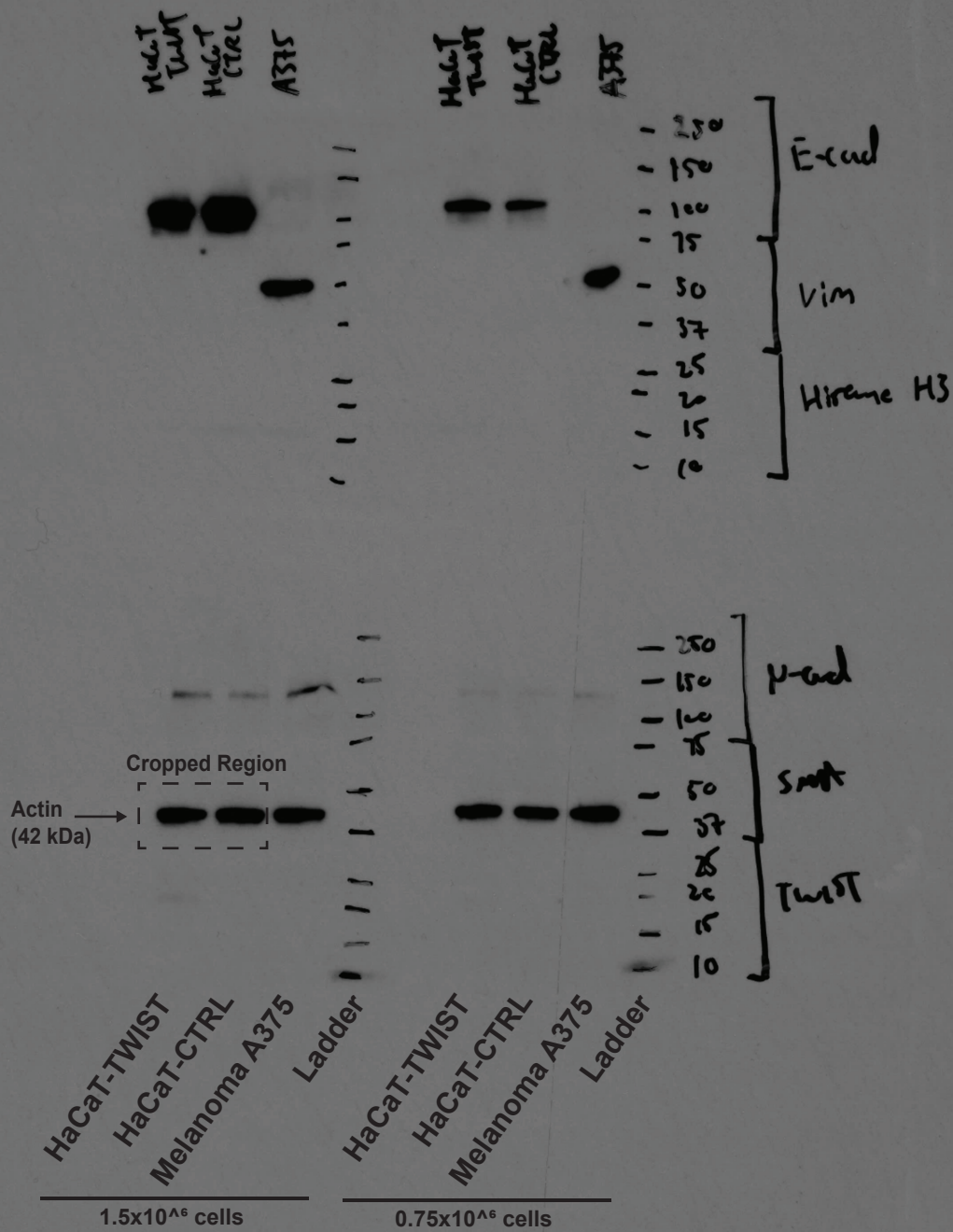

Supplement: Figure 4—source data 1. [file elife-101974-fig4-data1.zip › Figure_4C_Source_Data_1_Actin_labled.pdf]

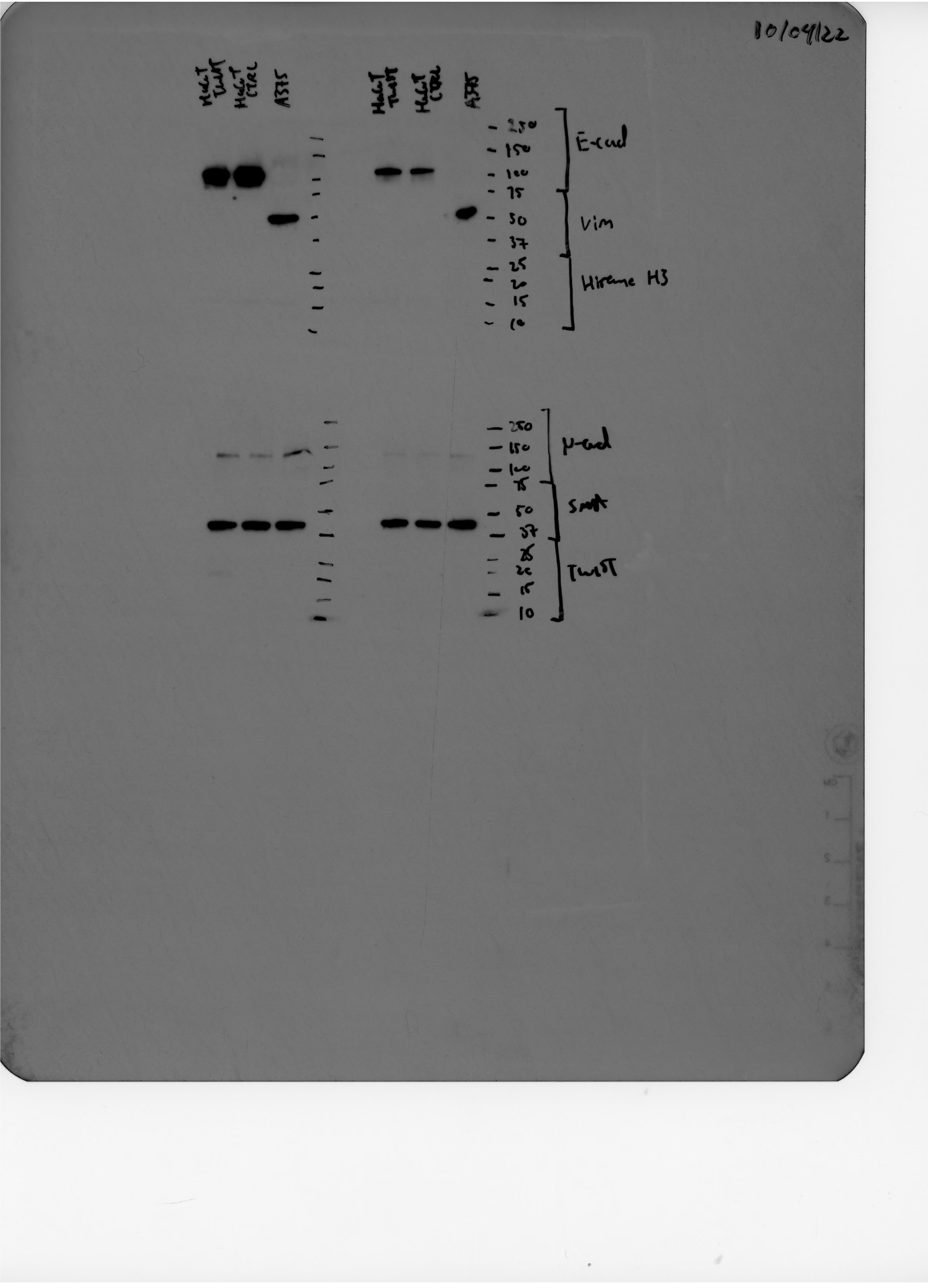

Supplement: Figure 4—source data 2. [file elife-101974-fig4-data2.zip › Figure_4C_Actin_Raw.png]

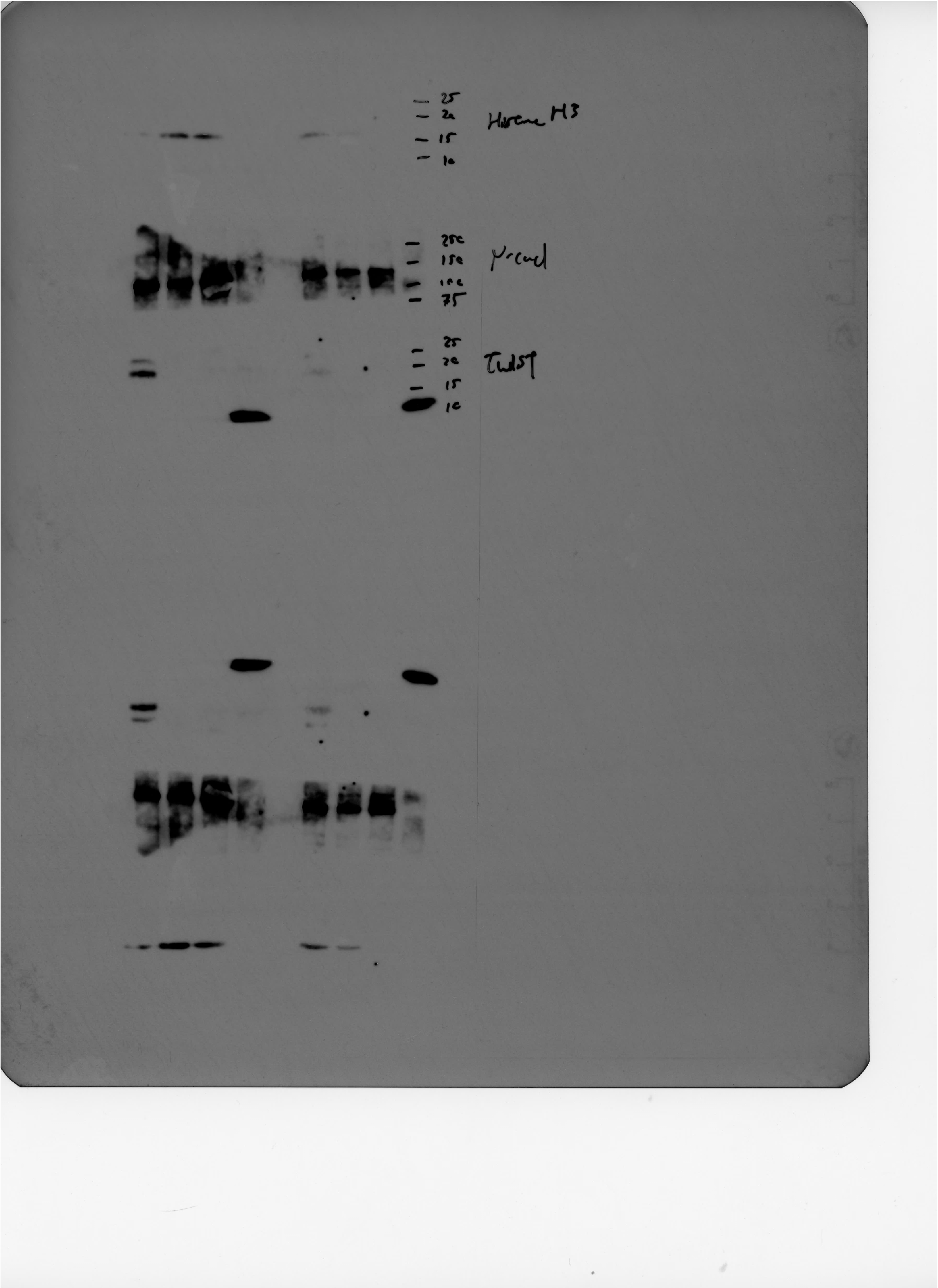

Supplement: Figure 4—source data 2. [file elife-101974-fig4-data2.zip › Figure_4C_TWIST_Raw.png]
