# Supplementary material for: Restraint of melanoma progression by cells in the local skin environment
Source: eLife. 2026 Jun 30;13:RP101974. doi: 10.7554/eLife.101974 (PMC13318302; doi:10.7554/eLife.101974)
Supplement: Peer review history

# Editor report: eLife Assessment

**DOI:** 10.7554/eLife.101974.3.sa0

**Authors:** Filippo Del Bene

**Affiliations:** Institut de la Vision France

**Keywords:** Convincing, Important

In this **important** study, the authors used a zebrafish model and scRNAseq analysis to show that a subset of keratinocytes within melanoma microenvironment highly up-regulate Twist and undergo Epithelial-Mesenchymal Transition (EMT). Surprisingly, when overexpressing Twist in keratinocytes, the resulting alteration in keratinocytes is inhibitory for melanoma invasion in both zebrafish and human cell culture models. The results are supported by **convincing** experimental data that provide new insights into the interactions between melanoma cells and their environment.

# Referee report: Reviewer #1 (Public review)

**DOI:** 10.7554/eLife.101974.3.sa1

Summary:

Ma et al. show that melanoma cells induce an EMT-like state in nearby keratinocytes and that when this state is induced experimentally by Twist-overexpression the resulting alteration in keratinocytes is inhibitory for melanoma invasion. These conclusions are based on experiments in vivo with zebrafish and, in vitro, with human cells. The work is carefully done and provides new insights into the interactions between melanoma cells and their environment.

Strengths:

Use of both zebrafish and human cells adds confidence that findings are relevant to human melanomas while also further demonstrating utility of the zebrafish system for discovering important new features of melanoma biology that could ultimately have clinical impacts. The work also combines a nice suite of approaches including different models for induced melanomagenesis in zebrafish, single cell RNA-sequencing, and more. Some of the final observations are intriguing as well, especially the possibility of EMT induced melanocyte-keratinocyte interactions via Jam3 expression; it will be interesting to see if these is indeed a mechanism for restraining melanoma invasion. The paper is clearly written and the inferences appropriate for the results obtained. Overall the work makes a solid contribution to our understanding of important, but too often neglected, roles of the tumor microenvironment in promoting or inhibiting tumor progression and outcome.

Weaknesses:

No critical weaknesses noted.

Comments on revisions:

The authors have adequately addressed my comments and concerns.

# Referee report: Reviewer #2 (Public review)

**DOI:** 10.7554/eLife.101974.3.sa2

Summary:

Manuscript by Ma et. al. utilizes a zebrafish melanoma model, single-cell RNA sequencing (scRNA-seq), a mammalian in vitro co-culture system, and quantitative PCR (Q-PCR) gene expression analysis to investigate the role keratinocytes might play within the melanoma microenvironment. Convincing evidence is presented from scRNA-seq analysis showing that a small cluster of melanoma-associated keratinocytes upregulate the master EMT regulator, transcription factor, Twist1a. To investigate how Twist-expressing keratinocytes might influence melanoma development, the authors use an in vivo zebrafish model to induce melanoma initiation while overexpressing Twist in keratinocytes through somatic transgene expression. This approach reveals that Twist overexpression in keratinocytes suppresses invasive melanoma growth. Using a complementary in vitro human cell line co-culture model, the authors demonstrate reduced migration of melanoma cells into the keratinocyte monolayer when keratinocytes overexpress Twist. Further scRNA-seq analysis of zebrafish melanoma tissues reveal that, in the presence of Twist-expressing keratinocytes, subpopulations of melanoma cells show altered gene expression, with one unique melanoma cell cluster appearing more terminally differentiated. The authors use computational methods to predict putative receptor-ligand pairs that might mediate the interaction between Twist-expressing keratinocytes and melanoma cells. Finally the authors established that similar keratinocyte phentypical changes also occurs in human melanoma tissues, setting a scene for future clinically relevant studies.

Strengths:

The scRNA-seq approach reveals a small proportion of keratinocytes undergoing EMT within melanoma tissue. The use of a zebrafish somatic transgenic model to study melanoma initiation and progression provides an opportunity to manipulate host cells within the melanoma microenvironment and evaluate their impact on tumour progression. Solid data demonstrate that Twist-expressing keratinocytes can constrain melanoma invasive development in vivo and reduce melanoma cell migration in vitro, establishing that Twist-overexpressing keratinocytes can suppress at least one aspect of tumour progression. Using GeoMX spatial transcriptomics platform to interrogate a series of early melanoma precursor lesions, enabled the authors to demonstrate similar EMT phenotype in keratinocytes also occurs in humans.

Weaknesses:

Due to limitations of the current model, no EMT marker gene expression was examined in melanoma tissue sections to determine the proportion and localization of Twist+ve keratinocytes within the melanoma microenvironment. However the authors compensated this through using spatial transcriptomics platform to interrogate a series of early melanoma precursor lesions in humans.

Due to technical limitations, it remain to be determined whether blocking EMT through down-regulation of Twist in keratinocytes may influence melanoma development.

Due to technical limitations, none of the gene expression changes detected through Q-PCR or scRNA-seq were examined using immunostaining or in situ hybridization, hence cellular resolution spatial information is lacking.

Overall, the data presented in this report draw attention to a less-studied host cell type within the tumour microenvironment, the keratinocytes, which, similar to well-studied immune cells and fibroblasts, could play important roles in either promoting or constraining melanoma development. Counterintuitively, the authors show that Twist-expressing EMT keratinocytes can constrain melanoma progression. While the detailed mechanisms remain to be uncovered, this is an exciting new line of research that warrant future studies.

Comments on revisions:

The authors have provided additional evidence to support their original conclusions, and the inclusion of spatial transcriptomic analysis using human samples strengthens the study. I did not identify any further issues that require attention.

# Referee report: Reviewer #3 (Public review)

**DOI:** 10.7554/eLife.101974.3.sa3

Summary:

In this study the authors use the zebrafish model and in vitro co-cultures with human cell lines, to study how keratinocytes modulate the early stages of melanoma development/migration. The authors demonstrate that keratinocytes undergo an EMT-like transformation in the presence of melanoma cells which lead to a reduction in melanoma cell migration. This EMT transformation occurs via Twist; and resulted in an improvement in OS in zebrafish melanoma models. Authors suggest that the limitation of melanoma cell migration by Twist-overexpressing keratinocytes was through altered cell-cell interactions (Jam3b) that caused a physical blockage of melanoma cell migration.

Strengths:

Authors describe a new cross-talk between melanoma and its major initial microenvironment: the keratinocytes and how instructed by melanoma cells keratinocytes undergo an EMT transformation, which then controls melanoma migration.

Overall, the paper is very well written, and the results are clearly organized and presented.

Weaknesses:

(1) To really show their last point it would be important to CRISPR KO Jam3b in melanoma with twist OE keratinocytes, in vivo or in vitro.

(2) Use of patient biopsies from early-stage melanomas vs healthy tissue to assess if there is a similar alteration of morphology of adjacent keratinocytes and increase in vimentin in human samples would strengthen the author's findings.

(3) Characterise better the cell-cell junctions and borders between cells (melanoma/ keratinocytes) with cellular and sub-cellular resolution. Since melanocytes can "touch" with their dendrites ~40 keratinocytes - can authors expand and explain better their model? Can this explain that in some images we cannot observe a direct interface between the cells?

Comments on revisions:

The authors answered most of the concerns raised.

# Author comment: Author response

**DOI:** 10.7554/eLife.101974.3.sa4

**Authors:** Yilun Ma, Mohita Tagore, Miranda V Hunter, Ting-Hsiang Huang, Emily Montal, Joshua M Weiss, Yingxiao Shi, Tuulia Vallius, Peter K Sorger, Richard M White

**Affiliations:** Weill Cornell Graduate School of Medical Sciences New York United States; Memorial Sloan Kettering Cancer Center New York United States; Memorial Sloan Kettering Cancer Center New York United States; Memorial Sloan Kettering Cancer Center New York United States; Memorial Sloan Kettering Cancer Center New York United States; Memorial Sloan Kettering Cancer Center New York United States; Harvard Medical School Boston United States; Harvard Medical School Boston United States; Harvard Medical School Boston United States; University of Oxford Oxford United Kingdom

The following is the authors’ response to the original reviews.

**Public Reviews:**

**Reviewer #1 (Public review):**

Summary:

Ma et al. show that melanoma cells induce an EMT-like state in nearby keratinocytes and that when this state is induced experimentally by Twist-overexpression the resulting alteration in keratinocytes is inhibitory for melanoma invasion. These conclusions are based on experiments in vivo with zebrafish and, in vitro, with human cells. The work is carefully done and provides new insights into the interactions between melanoma cells and their environment.

We appreciate your support for our overall conclusions.

Strengths:

The use of both zebrafish and human cells adds confidence that findings are relevant to human melanomas while also further demonstrating the utility of the zebrafish system for discovering important new features of melanoma biology that could ultimately have clinical impacts. The work also combines a nice suite of approaches including different models for induced melanomagenesis in zebrafish, single-cell RNA-sequencing, and more. Some of the final observations are intriguing as well, especially the possibility of EMT-induced melanocyte-keratinocyte interactions via Jam3 expression; it will be interesting to see if this is indeed a mechanism for restraining melanoma invasion. The paper is clearly written and the inferences are appropriate for the results obtained. Overall the work makes a solid contribution to our understanding of important, but too often neglected, roles of the tumor microenvironment in promoting or inhibiting tumor progression and outcome.

Weaknesses:

No critical weaknesses were noted.

**Reviewer #2 (Public review):**

Summary:

The manuscript by Ma et. al. utilizes a zebrafish melanoma model, single-cell RNA sequencing (scRNA-seq), a mammalian in vitro co-culture system, and quantitative PCR (Q-PCR) gene expression analysis to investigate the role keratinocytes might play within the melanoma microenvironment. Convincing evidence is presented from scRNA-seq analysis showing that a small cluster of melanoma-associated keratinocytes upregulates the master EMT regulator, transcription factor, Twist1a. To investigate how Twist-expressing keratinocytes might influence melanoma development, the authors use an in vivo zebrafish model to induce melanoma initiation while overexpressing Twist in keratinocytes through somatic transgene expression. This approach reveals that Twist overexpression in keratinocytes suppresses invasive melanoma growth. Using a complementary in vitro human cell line co-culture model, the authors demonstrate reduced migration of melanoma cells into the keratinocyte monolayer when keratinocytes overexpress Twist. Further scRNA-seq analysis of zebrafish melanoma tissues reveals that in the presence of Twist-expressing keratinocytes, subpopulations of melanoma cells show altered gene expression, with one unique melanoma cell cluster appearing more terminally differentiated. Finally, the authors use computational methods to predict putative receptor-ligand pairs that might mediate the interaction between Twist-expressing keratinocytes and melanoma cells.

Strengths:

The scRNA-seq approach reveals a small proportion of keratinocytes undergoing EMT within melanoma tissue. The use of a zebrafish somatic transgenic model to study melanoma initiation and progression provides an opportunity to manipulate host cells within the melanoma microenvironment and evaluate their impact on tumour progression. Solid data demonstrate that Twist-expressing keratinocytes can constrain melanoma invasive development in vivo and reduce melanoma cell migration in vitro, establishing that Twist-overexpressing keratinocytes can suppress at least one aspect of tumour progression.

Weaknesses:

While the scRNA-seq analysis of melanoma tissue and RT-PCR analysis of EMT gene expression in isolated keratinocytes provide evidence that a subpopulation of host keratinocytes upregulates Twist and other EMT marker genes and potentially undergoes EMT, the in vivo evidence for keratinocyte EMT within the melanoma microenvironment is based on cell morphology in a single image without detailed characterization and quantification. No EMT marker gene expression was examined in melanoma tissue sections to determine the proportion and localization of Twist+ve keratinocytes within the melanoma microenvironment.

We agree this needed better support. To address this, we have collaborated with the Sorger lab who has performed Spatial Transcriptomics on early human melanoma samples (n=8 samples). The advantage of this method is that they can dissect microregions of interest (MRs) RNA-seq to discern keratinocytes vs. melanocytes. We queried regions that had higher or lower numbers of atypical melanocytes in these biopsies with our TAK or TWIST signature. While the normal sample had no enrichment, we found that a subset of the human samples had evidence of these signatures in the keratinocytes, particularly the ones which had a higher proportion of atypical melanocytes. These data support our model that early melanomas enact an EMT like program in a subset of nearby keratinocytes.

The scRNA-seq UMAP suggests the proportion of EMT keratinocytes within the melanoma microenvironment is very small, raising questions about their precise location and significance within the tumour microenvironment. Although both in vivo and in vitro evidence demonstrates that Twist-expressing keratinocytes can suppress melanoma progression, the conditions modelled by the authors involve over-expression of Twist in all keratinocytes, which do not naturally occur within the melanoma microenvironment and, therefore, might not be relevant to naturally occurring melanoma progression. The author did not test whether blocking EMT through down-regulation of Twist in keratinocytes may influence melanoma development, which would establish the role of Twist expression keratinocytes in the melanoma microenvironment.

We entirely agree, and ideally would do the exact experiment you suggested, which is to knockout TWIST in the keratinocytes using CRISPR and see how this affects the tumor phenotype. However, despite our best efforts, we do not yet have an efficient method for performing knockouts in the tumor microenvironment. If we used standard 1-cell embryo transgenic approaches with a krt4-Cas9, this would severely disrupt skin development in the whole animal, and would be viable. Theoretically, we could do this with TEAZ, but we have found that the expression of Cas9 in the microenvironment (i.e. under a krt4 promoter) is relatively inefficient. For example, we tried a krt4-Cas9 coupled with an sgRNA against GFP (as a test of the system) and this did not work well. Thus, a major goal for future studies is to develop a technology that would allow us to do this exact experiment. Finally, we do not have enough cells present in the sections to answer the question of whether the EMT keratinocytes are associated with certain melanoma cell states (i.e. proliferative, invasive), although we agree this would be an important question for future studies.

To address the potential mechanism by which Twist-expressing keratinocytes suppress melanoma progression, a second scRNA-seq analysis was conducted. However, this analysis is not adequately presented to provide strong evidence for proposed mechanisms for how Twist-expressing keratinocytes suppress melanoma cell invasion. CellChat analysis was used to attempt to identify receptor-ligand pairs that might mediate keratinocyte-melanoma cell interaction, but the interactions between tumour-associated keratinocytes (TAK) and melanoma cells were not included in the analysis. Furthermore, although genetic reporters were used to label both keratinocytes and melanoma cells, no images showing the detailed distribution and positional information of these cells within melanoma tissue are presented in the report. None of the gene expression changes detected through Q-PCR or scRNA-seq were validated using immunostaining or in situ hybridization.

As noted above, we have now added human biopsy samples from the Sorger lab to our analysis, showing that the TAK/TWIST keratinocytes occur directly adjacent to the atypical melanocytes in these samples. While these early melanomas are quite difficult to obtain (most samples are used for diagnostic purposes), this provides further support to our zebrafish models.

Overall, the data presented in this report draw attention to a less-studied host cell type within the tumour microenvironment, the keratinocytes, which, similar to well-studied immune cells and fibroblasts, could play important roles in either promoting or constraining melanoma development.

Counterintuitively, the authors show that Twist-expressing EMT keratinocytes can constrain melanoma progression. While the detailed mechanisms remain to be uncovered, this is an interesting observation.

**Reviewer #3 (Public review):**

Summary:

In this study the authors use the zebrafish model and in vitro co-cultures with human cell lines, to study how keratinocytes modulate the early stages of melanoma development/migration. The authors demonstrate that keratinocytes undergo an EMT-like transformation in the presence of melanoma cells which leads to a reduction in melanoma cell migration. This EMT transformation occurs via Twist; and resulted in an improvement in OS in zebrafish melanoma models. Authors suggest that the limitation of melanoma cell migration by Twist-overexpressing keratinocytes was through altered cell-cell interactions (Jam3b) that caused a physical blockage of melanoma cell migration.

Strengths:

The authors describe a new cross-talk between melanoma and its major initial microenvironment: the keratinocytes and how instructed by melanoma cells keratinocytes undergo an EMT transformation, which then controls melanoma migration. Overall, the paper is very well written, and the results are clearly organized and presented.

Weaknesses:

(1) To really show their last point it would be important to CRISPR KO Jam3b in melanoma with twist OE keratinocytes, in vivo or in vitro.

The CellChat data suggest that Jam3b is likely important in melanoma development, as it has been shown to be important in melanocyte development (Eom, Dev Biol 2021). Studying this specifically in melanoma progression is an area of ongoing study in our lab, and we have begun to generate the Jam3b knockouts as you suggested. Since this set of experiments is quite extensive, we feel this set of data deserves a separate manuscript, which we hope to complete in the near future.

(2) The use of patient biopsies from early-stage melanomas vs healthy tissue to assess if there is a similar alteration of morphology of adjacent keratinocytes and an increase in vimentin in human samples would strengthen the author's findings.

As noted above, we have now added human biopsy samples from the Sorger lab to our analysis, showing that the TAK/TWIST keratinocytes occur directly adjacent to the atypical melanocytes in these samples. While these early melanomas are quite difficult to obtain (most samples are used for diagnostic purposes), this provides further support to our zebrafish models.

(3) The cell-cell junctions and borders between cells (melanoma/ keratinocytes) should be characterized better, with cellular and sub-cellular resolution. Since melanocytes can "touch" with their dendrites ~40 keratinocytes - can authors expand and explain better their model? Can this explain that in some images we cannot observe a direct interface between the cells?

We have now added higher resolution images of these junctions. Our overall hypothesis, related to point (2) above, is that Jam3b mediates these junctions between melanoma cells and keratinocytes, which is why we are now pursuing this in a followup study.

**Recommendations for the authors:**

**Reviewer #1 (Recommendations for the authors):**

(1) Please say a little more about any phenotypes that might have been evident inTwist-overexpression fish in the absence of melanomas, and clarify in the text that these were mosaic animals, as a first (incorrect) reading left the impression that stablelines had been made.

In these experiments, we co-injected the melanoma plasmids along with the krt4-TWIST plasmids, creating mosaic animals. Because of this, we did not have a way of specifically looking at the effect of TWIST in the absence of melanoma. We agree this needs better clarification and have added this to the Results.

(2) Violin plot colors in main and Supplementary Figures tend to obscure data points. Colors for keratinocyte clusters are not discernible in Figure 4C.

We have remade the plots in a different color scheme to try and make these stand out more easily.

(3) Clarify that N-cadherin = cdh2 in Figure 1

We have fixed this in the legend for Figure 1.

(4) Clarify the relationship between keratinocytes highlighted in Figure 2B and used for Hallmark expression in Figure 2B, and those analyzed for expression of candidate genes in Figure 2E. The last shows many NKC whereas whereas even the larger group circled in Figure 2B as keratinocytes seems to have far fewer cells, unless massively overplotted. Is the rest of that cluster in Fig. 2B keratinocytes as well?

In the analysis in Figure 2E, we first calculated genes differentially expressed in the TAK vs. NKCs (found in Figure 2B). We used those genes as input into GSEA analysis, which showed enrichment for EMT programs specifically in the TAKs. We recognize that the number of TAKs is relatively small (compared to all of the other cells in the single-cell UMAP) but that is the most we were able to get from this particular scRNA run, because the melanoma cells naturally make up the vast majority of the cells in the 10X run. This is why we performed downstream mechanistic analysis (in the rest of the paper) to ensure this result was not an artifact of a small number of TAKs.

(5) Define "NES" in the Figure 2 legend.

NES indicates “Normalized Enrichment Score”, a standard output of GSEA. This has been added to the legend.

(6) Indicate how many control vs. Twist+ fish were found to have invasive vs non-invasive tumors upon histological examination. Were tumors in the latter fish always contained within the epidermis proper, or did some extend deeper if given enough time?

In the histology analysis, we used n=3 control fish and n=3 TWIST overexpressing fish. Main Figure 3 shows n=1 of these fish from each group, and the other n=2 from each is shown in Supplemental Figure 1. In this cohort (taken at 26 weeks), all of the TWIST tumors were contained within the epidermis, but we did not let them grow longer to see if (given enough time) they could have invaded below this. Around 26 weeks, the survival decreased so made this an unfeasible experiment at later time points. We have added a statement about this to the Results section.

**Reviewer #2 (Recommendations for the authors):**

Going through the data presented in the figures, here are my comments:

(1) Figure 1: To strengthen the evidence that keratinocytes in the melanoma microenvironment undergo EMT, it would be beneficial to provide immunostaining or in situ data for EMT marker genes within melanoma tissue sections co-stained with a keratinocyte marker (such as an anti-GFP antibody).

We agree this type of analysis is an important validation of our findings. Doing this in zebrafish tumors is difficult, as human/mouse antibodies for EMT marker genes typically do not work in fish. In addition, we felt that validating our results in human melanomas would make our findings more generalizable. Therefore, we established a collaboration with Peter Sorger’s lab, who have been performing high-resolution spatial transcriptomics on early melanoma samples from humans. While these are difficult to attain (since most early lesions are processed for clinical diagnosis) they have a collection of n=8 samples that they subjected to GeoMX spatial analysis. In this method, the samples are first stained with antibodies to definitively mark keratinocytes (PANCK) vs. melanoma cells (SOX10) and all samples are reviewed by expert pathologists. From this, microregions (MRs) of interest are selected to then undergo RNA-seq. After control analysis to ensure both keratinocytes and melanocytes were present in the samples, they then used our TAK or TWIST signatures as a query. Both signatures were enriched in the keratinocytes adjacent to early melanomas, but not in normal skin samples or in samples with few atypical melanocytes. This provides further evidence that the altered keratinocytes we see in our fish are present and enriched in human biopsy specimens.

(2) Figure 2: In panel B, the UMAP shows the separation of single cells, and keratinocytes are circled. However, there are two clusters of keratinocytes, and the graph does not indicate which cluster represents tumour-associated keratinocytes (TAKs) versus normal keratinocytes (NKCs). The two clusters also appear to differ in abundance, so it would be helpful to report the proportion of keratinocytes that are TAKs undergoing EMT, according to the individual dots in Figure 2E. In Figure 2E,TAKs seem to have very few cells compared to the other clusters. Given the relatively small number of EMT-TAKs detected in the single-cell RNA-seq data, I wonder how much direct influence these cells could exert on the bulk of melanoma cells in vivo.The evidence would be strengthened if an IHC analysis could show the location of Twist-expressing keratinocytes within the melanoma microenvironment and whether they are associated with certain melanoma cell markers but not others (i.e., markers indicating different differentiation states of melanoma cells). To further support the role of Twist-expressing keratinocytes in the melanoma microenvironment, it would be beneficial to perform a knockout (KO) of Twist in keratinocytes within the melanoma microenvironment.

In Figure 2B, we agree that the color scheme made it difficult to discern TAKs vs. NKCs.

We have changed the color scheme to make this more clear.

The number of TAKs undergoing EMT is relatively small, and this is why we performed the overexpression studies of TWIST in order to expand the field of keratinocytes undergoing EMT. To get at the question of whether these are really important in tumor initiation and progression, we ideally would do the exact experiment you suggested, which is to knockout TWIST in the keratinocytes using CRISPR and see how this affects the tumor phenotype. However, despite our best efforts, we do not yet have an efficient method for performing knockouts in the tumor microenvironment. If we used standard 1-cell embryo transgenic approaches with a krt4-Cas9, this would severely disrupt skin development in the whole animal, and would not be expected to be viable. Theoretically, we could do this with TEAZ, but we have found that the expression of Cas9 in the microenvironment (i.e. under a krt4 promoter) is relatively inefficient. For example, we tried a krt4-Cas9 coupled with an sgRNA against GFP (as a test of the system) and this did not work well. Thus, a major goal for future studies is to develop a technology that would allow us to do this exact experiment. Finally, we do not have enough cells present in the sections to answer the question of whether the EMT keratinocytes are associated with certain melanoma cell states (i.e. proliferative, invasive), although we agree this would be an important question for future studies.

(3) Figure 4: Co-culture results show that melanoma cells migrate further on a control HaCaT cell monolayer compared to a TWIST-overexpressing HaCaT cell monolayer. While this phenotype might support the conclusion that TWIST-expressing keratinocytes reduce melanoma cell invasion, it should be interpreted with caution. The data can be interpreted as TWIST-HaCaT cells inhibiting melanoma cell migration; however, an alternative explanation cannot be ruled out. For example, wild-type HaCaT cells might provide a suitable substrate for melanoma cells to migrate, whereas TWIST-HaCaT cells lack this property. To address this, the baseline melanoma cell migration should be established in this assay by coating the plate with cells from the same melanoma cell line and allowing melanoma cells from the flipped cover slip to migrate out.

We have performed the experiment you suggested using Hs.294T and SKMEL2 cells and provided this as a new Supplemental Figure 2. This demonstrated that the melanoma cells in this context could indeed migrate out of the coverslip at baseline. Thus, it is possible, as you indicated, that the phenotype we have observed might be due to something lacking in the TWIST keratinocytes that promotes migration. Since we cannot differentiate between these two possibilities (i.e. that TWIST KCs actively inhibit migration vs. lacking something that promotes migration), we have modified the text to indicate both of these possible mechanisms could be at play.

(4) In the representative images shown in the figure, it appears that both HaCaT cells and melanoma cells in the upper and lower panels are at very different densities."Contact inhibition" and "cell sorting" are well-known phenomena in tissue-cultured cells, so when cells are seeded at different densities, their ability to move away from the initial location could vary. From the Materials and Methods section, it is unclear why cell densities are drastically different in the images presented. Images in the upper panel show both melanoma cells and keratinocytes at lower densities, and in the TWIST group, melanoma cells under the cover slip appear to aggregate into clusters with TWIST-expressing keratinocytes surrounding each aggregated cluster. This suggests that cell sorting might be occurring, potentially mediated by cadherins or Eph-ephrins.

We recognized this discrepancy as well. In the setup of the experiment, we seeded the exact same number of cells for both the Hs.294T (Figure 4E) and SKMEL2 (Figure 4G) experiment. But when we took the images after 20 hours of co-culture, it was clear that the HaCat densities were different, as seen in the figures. We suspect this might be because these two melanoma cells may secrete different factors (i.e. growth factors) that impact upon HaCat proliferation, adhesion or cell sorting. Despite this, in terms of the ability of the melanoma cells to migrate into the HaCATs, we saw similar results across both experiments, suggesting that it is not HaCAT density alone that explains the results. But we agree we need to clarify this point about cell density more clearly in the manuscript, and we have amended the Discussion to indicate the above points.

(5) Figure 5: Single-cell RNA-seq analysis comparing cells from control melanomas with cells from melanomas developed in a Twist-expressing keratinocyte background could provide valuable information on how melanoma cells alter their phenotype and how Twist-expressing keratinocytes respond to melanoma development. However, the information presented in the manuscript is not persuasive in this regard (appears to be minimal).

(a) In Figure 5C, the differences between melanoma cells in a control background versus those in a Twist-expressing keratinocyte background include cells from more than one unique cluster, but most of the different clusters are not discussed, except for one prominent cluster indicated by an arrow.

The reason we pointed out that one cluster is that it was the major thing that was different in the control melanomas vs. the TWIST melanomas. To better clarify this point, we have made a new Supplemental Figure 3 comparing the clusters in each situation: 7 in the control melanomas vs. 8 in the TWIST melanomas (Supp. Figure 3d). To then better understand the nature of the TWIST melanomas, we performed Gene Set Enrichment Analysis (GSEA) compared to the control melanomas. Interestingly, this revealed a striking enrichment for pathways related to oxidative phosphorylation using both GO and Hallmark terms. Because we had previously shown that melanoma cells with high ox-phos are typically in the more melanocytic and less invasive state (Lumaquin-Yin, Nature Communications 2023), we therefore analyzed our TWIST melanomas by comparing this unique cluster to the well-annotated melanoma cell state signatures from Tsoi et al (Cancer Cell, 2018). This showed that most of the TAKs and TWIST-KCs were in the melanocytic/transitory cluster, which are thought to be the least invasive of all the melanoma cell states. Thus, it seems likely that high levels of TWIST in the keratinocytes induces a low invasion state in the melanoma cells. We have added this data and interpretation to the Results and Discussion sections of the manuscript.

(b) In Figure 5D, it is unclear whether TAKs include both wild-type keratinocytes and Twist-expressing keratinocytes.

We oversimplified this plot for the sake of visualization, but realize that in doing so we obscured some important details. In the plot, we separate normal keratinocytes (NKCs) vs. tumor associated keratinocytes (TAKs). TAKs are, by definition, TWIST^hi^/EMT^hi^ and represent upregulation of endogenous TWIST. In contrast, when we force overexpression of TWIST in the keratinocytes, then we see an entirely new cluster appear, as expected.

(c) In Figure 5F, TAKs are interacting with melanoma cells so it is unclear why the CellChat analysis did not include TAKs.

This was an oversight on our part, and the Figure has now been corrected to include this. TAKs in both the control and TWIST melanomas have numerous interaction partners, whereas the TWIST-KCs have relatively fewer and more specific interactions.

(d) Finally, Figure 5G needs clearer labelling,currently unclear which gene is expressed by the sender and which is by the receiver.

This has been clarified in Figure 5F with specific indicators of “sender” vs. “receiver”.

**Reviewer #3 (Recommendations for the authors):**

(1) Figure 1E - in this figure, it is possible to observe the altered morphology of keratinocytes but these cells are not in the vicinity of the melanoma cells - can authors please make a zoom-in in the region of the interface? And quantify the distance between cells - at least the image they show looks like the cells that are mostly de-formed are far away from the melanoma but perhaps was just this example....please clarify. Or there are patches of keratinocytes that go through EMT and others that maintain their epithelial structure?

We have now added zoom-in images of the interface (Figure 1E). In nearly all sections examined, some keratinocytes maintain their hexagonal normal epithelial structure, but the majority of the cells appear altered. We have attempted to quantify this effect, along with the distance between cells with this EMT-like morphology, but have not found a reliable method given the heterogeneity across samples. That is why we instead chose to quantify the EMT-like keratinocytes (what we refer to as TAKs) using single-cell RNA seq, which showed that 32% of the population had the TAK signature, whereas 68% resembled normal keratinocytes. We feel this is more quantitative than imaging alone.

This data has been added to the Results section.

(2) Figure 3B - could not find the number of fish analyzed.

This was an oversight on our part. We studied n=135 control melanomas vs. n=118

TWIST melanomas. This data has now been added to Figure 3B.

(3) Figure 3D - missing a graph with quantification and zoom images in the tail keratinocytes/ melanoma interface.

In this particular cohort of animals, we unfortunately did not specifically track body vs. fin melanomas, so we are not able to quantify this.

(4) Figure 4 - it would be nice again to have a zoom-in to observe the interface of cells- maybe use a phalloidin staining to visualize better how cells are touching each other.

We have added a zoom in image of the interface to the image (Figure 4E). We have very much wanted to do immunohistochemistry (not just for phalloidin, but for other markers as well) on these coverslip co-cultures and have tried, but we have not been successful. This is likely because the assay requires plastic plates, which are incompatible with doing this, but agree that getting this to work would be an important area for future development.

(5) I believe the paper deserves a last figure - with the model.

We agree and this has now been added as Figure 7.
